# Supplementary material for: Decoding the Role of CD271 in Melanoma
Source: Cancers (Basel). 2020 Aug 31;12(9):2460. doi: 10.3390/cancers12092460 (PMC7564075; doi:10.3390/cancers12092460)
Supplement: Supplementary file 1 [file cancers-12-02460-s001.pdf]

cancers

MDPI

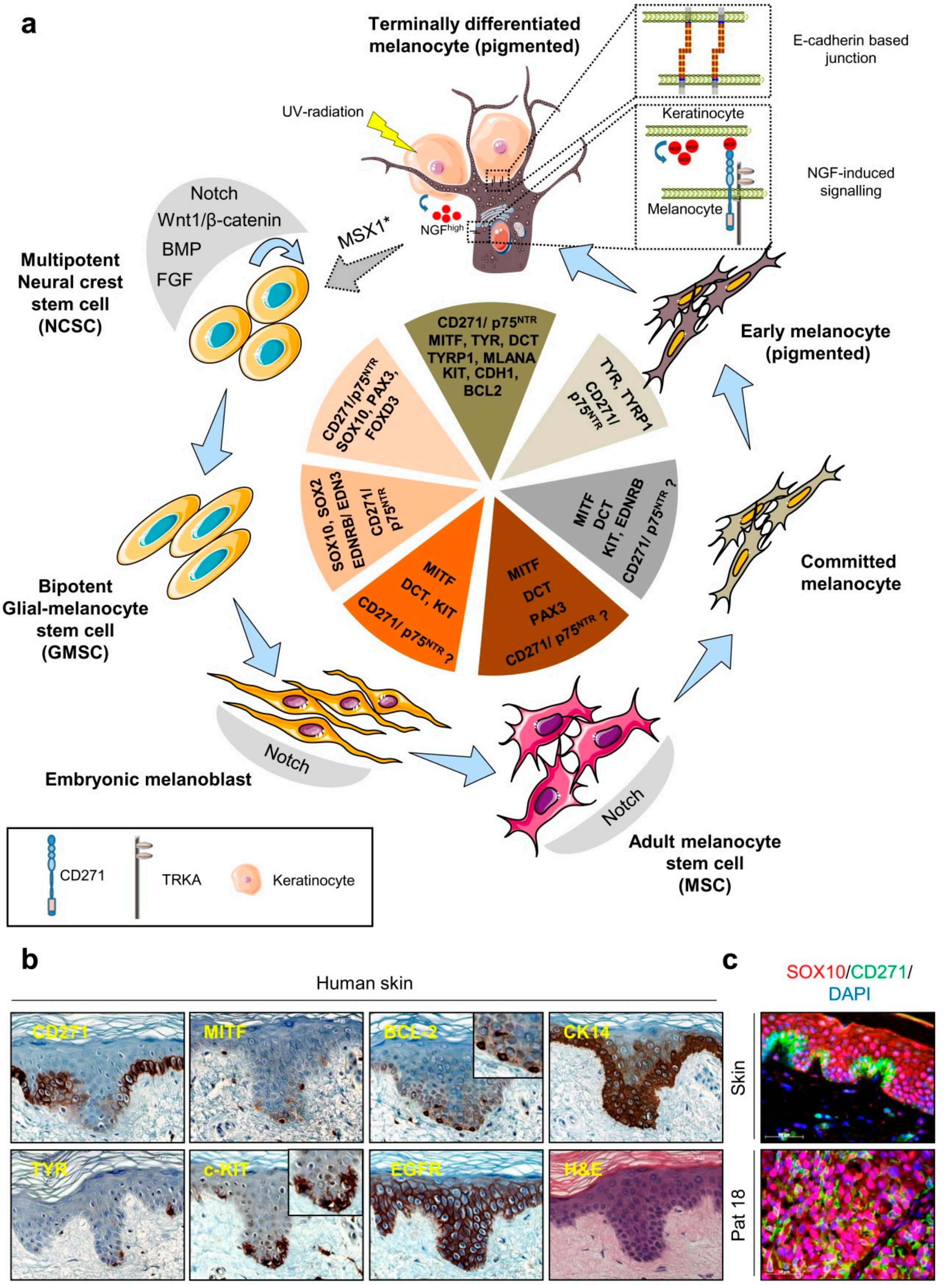

Figure S1. The evolution of melanocytes. (a) In a first step, the defined differentiation of CD271⁺ multipotent neural crest stem cells (NCSCs) that are maintained by a concerted activation of Notch/Wnt/BMP/FGF signaling gives rise to a CD271⁺ bipotent glial-melanocyte precursor (GMSC). The formation of embryonic melanoblasts is triggered by Wnt signaling (Wnt1/Wnt3) driving the expression of MITF (microphthalmia-associated transcription factor), DCT (dopachrome tautomerase), and KIT (c-Kit). Embryonic melanoblasts in turn give rise to adult melanocyte stem cells (MSCs). Notch signaling is required for the maintenance of melanoblasts and MSCs. The expression of KIT, EDNRB, MITF, and DCT drives the commitment of MSCs. Committed

melanocytes give rise to early and terminally differentiated melanocytes. The latter reside in the *stratum basale* of the skin in close proximity to keratinocytes, connected via the adherence junction protein E-cadherin (CDH1). In addition, melanocyte survival is supported by keratinocyte-secreted nerve growth factor (NGF), which in turn binds and activates TRKA/CD271-dependent signaling processes in melanocytes. The secretion of NGF by keratinocytes is triggered and promoted by UV radiation. Over expression of MSX1 induced the de-differentiation of melanocytes to NCSCs. Although the expression of CD271 in NCSCs and melanocytes was empirically evidenced, whether and to which extent the levels of CD271 change during the genesis of melanocytes is unknown (?). Schemes depicting melanocyte development were adapted and modified from references [21,22]. Cell images were taken from https://smart.servier.com/. (b) Immunohistochemistry of human skin showing the expression of CD271, MITF, BCL2, and CK14 (upper row) and TYR, c-KIT, and EGFR (lower row) in non-serial sections. H&E (hematoxylin&eosin) staining was used for a histological separation of keratinocytes and melanocytes. (c) CD271 is co-expressed with SOX10 in human skin and metastatic melanoma (Pat 18).

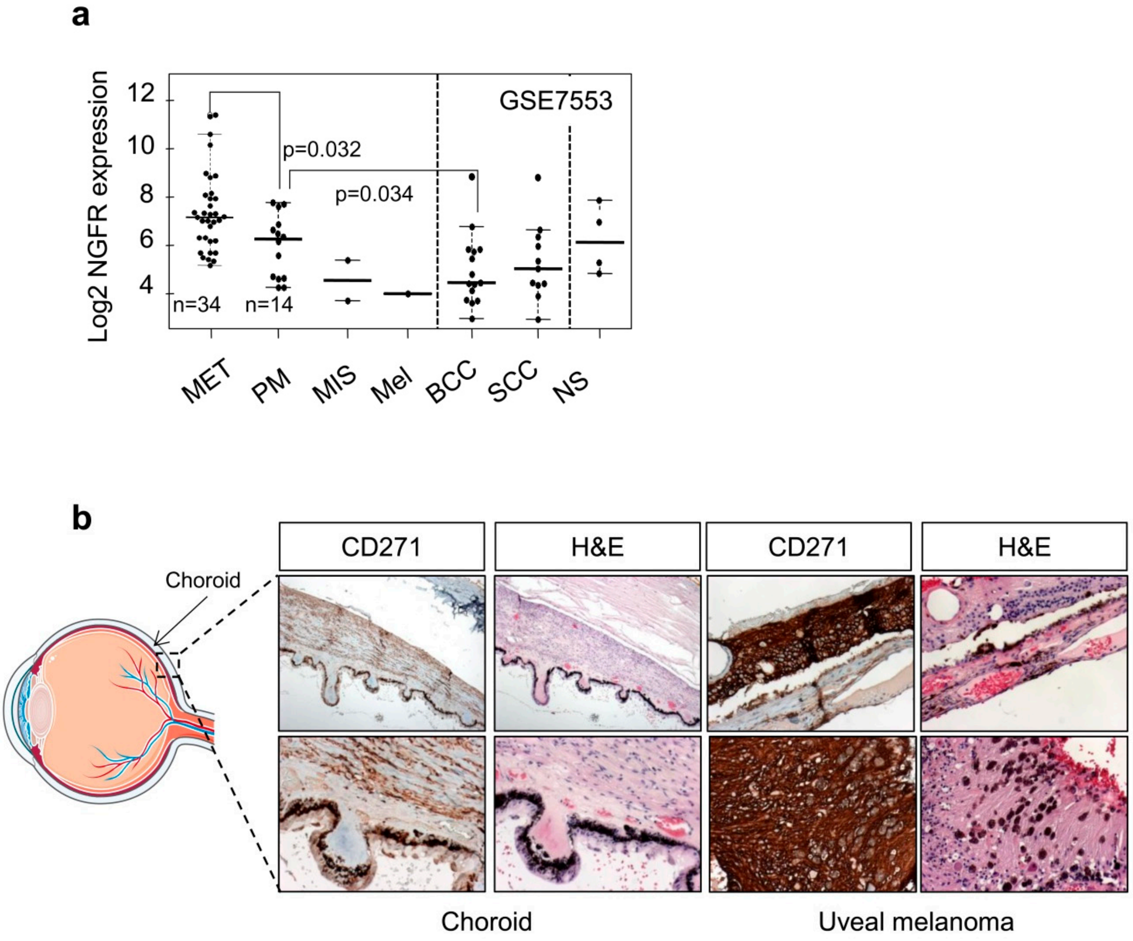

Figure S2. Expression of CD271 in skin and skin cancer. (**a**) Comparative representation of levels of NGFR expression among metastatic (MET) and primary (PM) melanoma, melanoma in-situ (MIS) and melanocytes with non-melanoma skin cancer subtypes basal cell carcinoma (BCC), squamous cell carcinoma (SCC) and normal skin (NS) of dataset GSE7553. Levels of CD271 was significantly higher in PM and MET than in BCC and SCC. **(b)** Expression of CD271 (red) and MITF (brown) within the hair root. **(c)** Comparative investigation of levels of CD271 in the choroid of the eye (left panels) and uveal melanoma (right panels). Overview images are shown in the first row; dark spots indicate melanin-rich areas/cells.

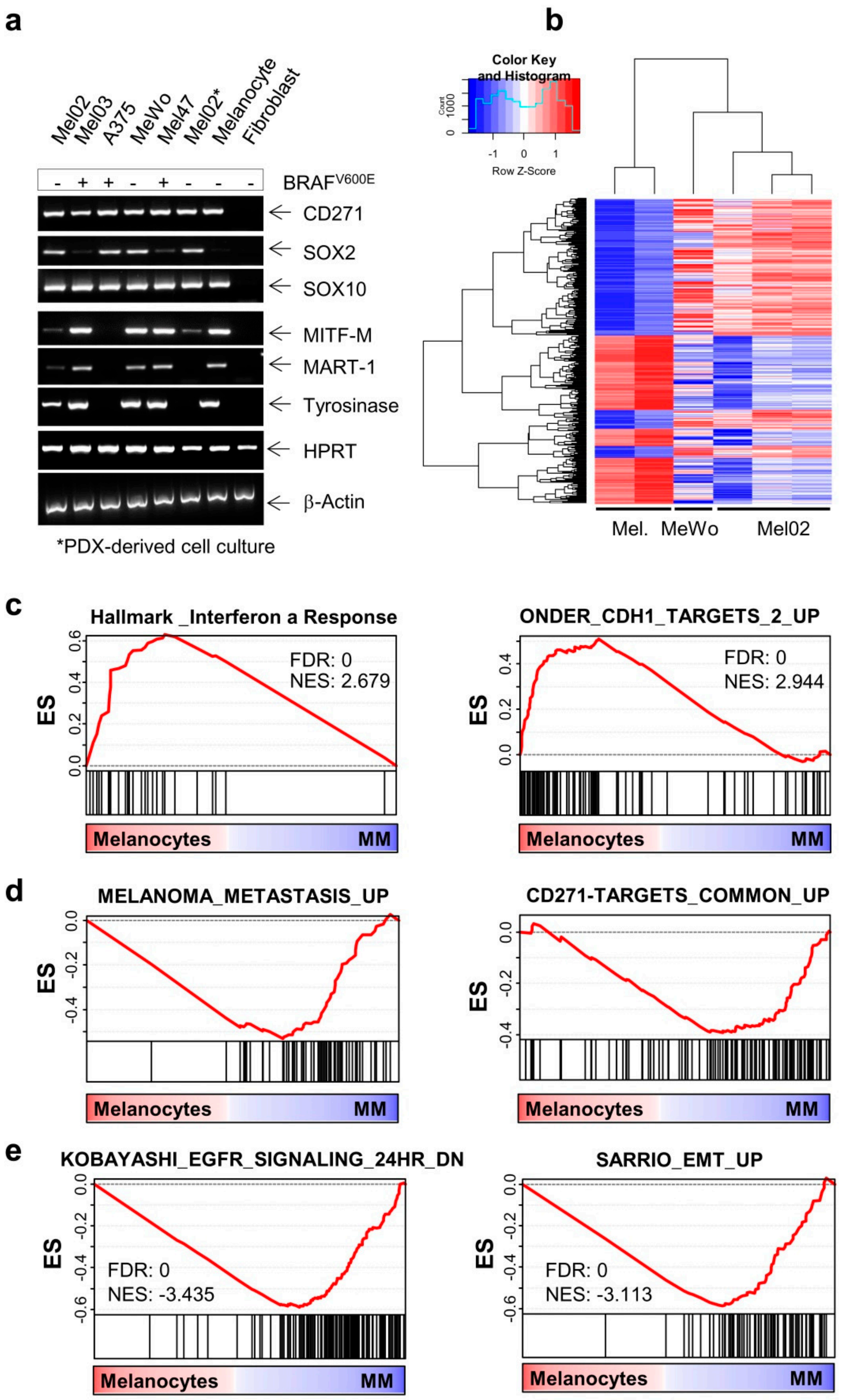

Figure S3. Molecular programs defining the transformation of melanocytes to metastatic melanoma. (**a**) The investigation of mRNA expression levels of CD271, SOX2, SOX10, MITF-M, MART1, and TYR by RT-PCR of cell lines derived from melanoma metastases with melanocytes and fibroblasts revealed distinct and overlapping expression. (**b**) Expression profiling of cell lines (MeWo; high passage and Mel02; low-passage) established from lymph nodes metastases with human adult melanocytes revealed ~2,000 differentially regulated genes. (**c**) Gene-set enrichment analysis (GSEA) of the latter revealed the predominance of genes associated with response to interferon A and

E-cadherin signaling in melanocytes and the predominance of genes associated with metastasis, CD271-dependent processes, suppression of EGFR-based signaling and EMT in metastatic melanoma cells.

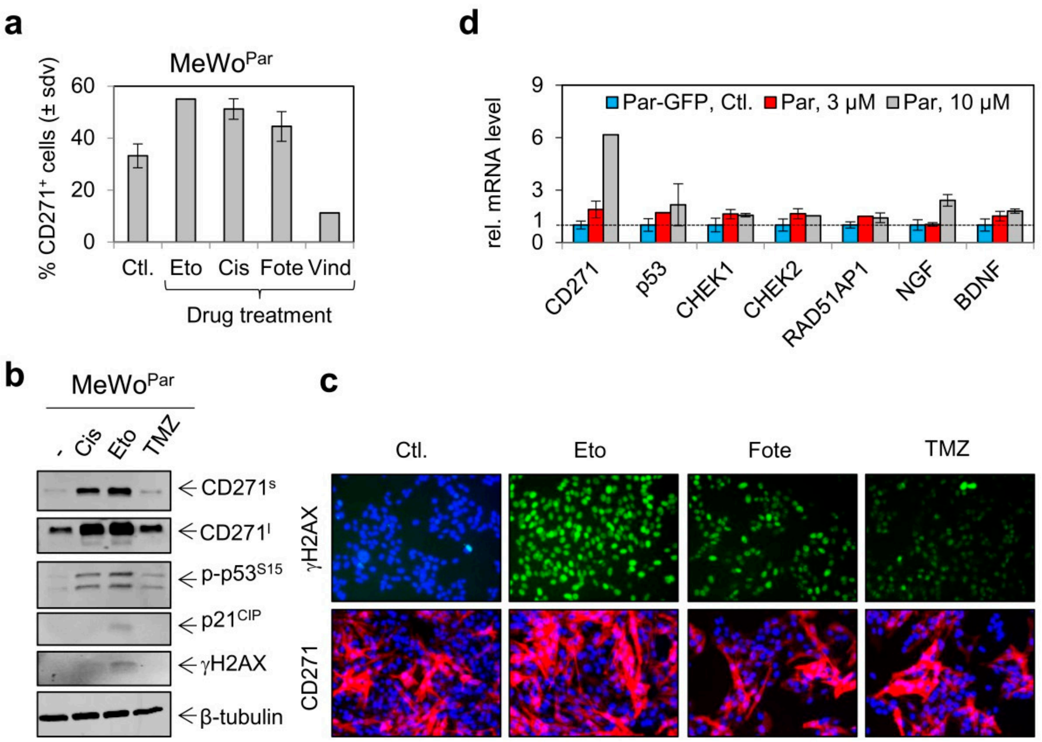

Figure S4. Chemotherapeutic drugs increase the expression CD271. (**a**) Flow cytometric analysis of cell surface levels of CD271 of MeWo cells, untreated (Ctl.) or treated for 24h with chemotherapeutic drugs etoposide (Eto, 10 µM), cisplatin (Cis, 10 µM), fotemustine (Fote, 30 µg/ml) or vindesine (Vind, 1 ng/ml). The percentage of CD271+ cells is shown, error bars indicate sdv. (**b**) Immunblot analysis of whole protein lysates of MeWo cells, untreated (-) or treated with Cis, Eto, and temozolomide (TMZ, 10 µM) for 48h. Levels of CD271 after short (s) or long (l) exposure time are shown. Eto most effectively induced the DNA-damage response. (**c**) Immunofluorescence microscopy of MeWo cells for phosphorylated (S139) histone 2A (γH2AX) and CD271 following treatment with Eto, Fote, and TMZ with indicated dosages after 24h. (**d**) Investigation of cisplatin-responsive genes in MeWo cells after Cis treatment (3, 10 µM) for 24h.

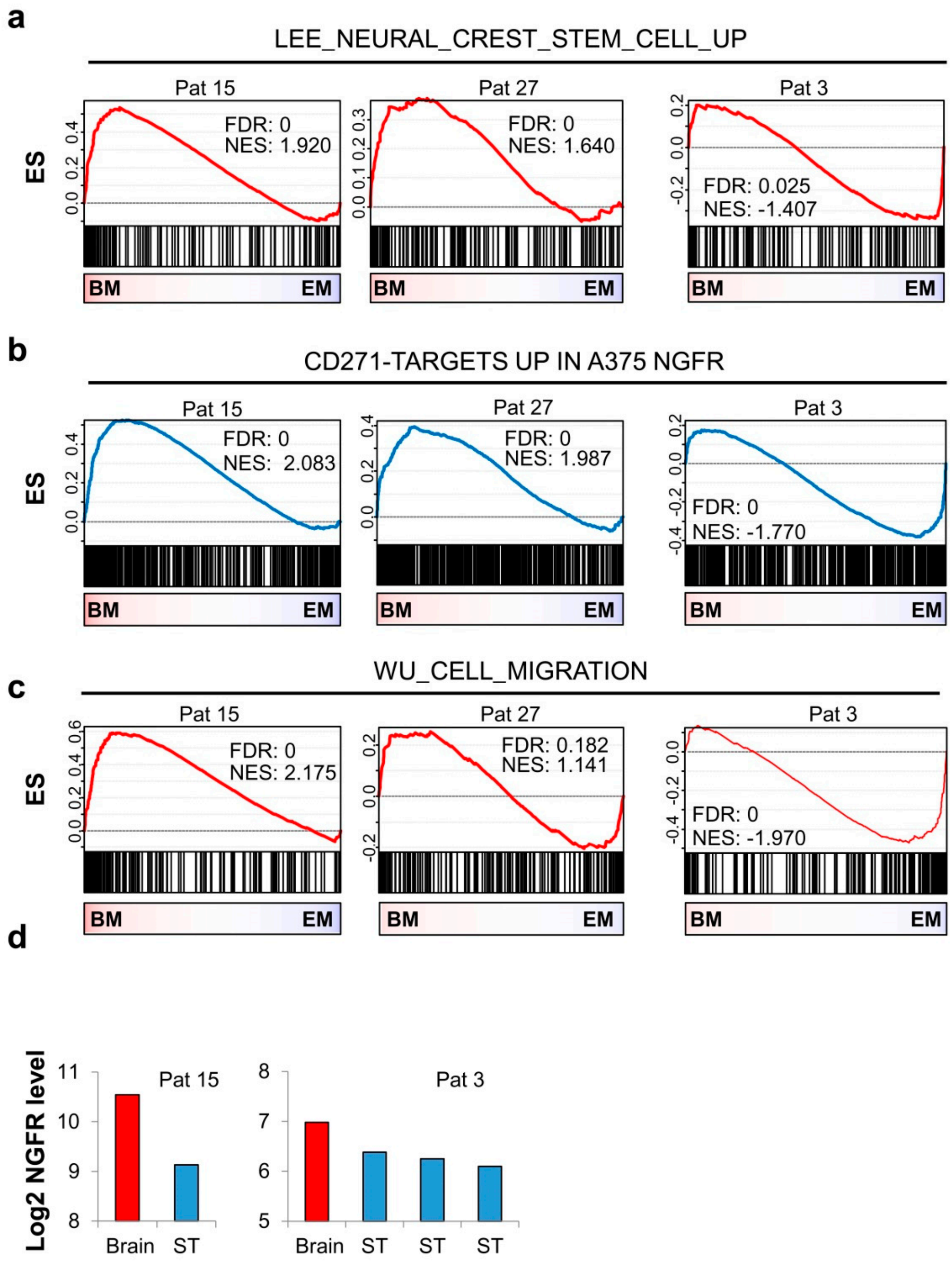

Figure S5. The expression of CD271 is sufficient for sub-clustering melanoma brain metastases. (**a**) GSEA of matched pairs of brain (BM) and extracranial (EM) metastases of dataset GSE50493 revealed prevalent expression of NCSC-specific genes in BM with concordantly high levels of (**b**) CD271-associated genes (Pat 15, 27) absent in a patient with CD271 negative tumors (Pat 3). (**c**) Genes potentially predicating a migratory cancer cell phenotype showed at least partially a comparable tendency. (**d**) Levels of CD271 expression in Pat 27 and Pat 3 tumors.

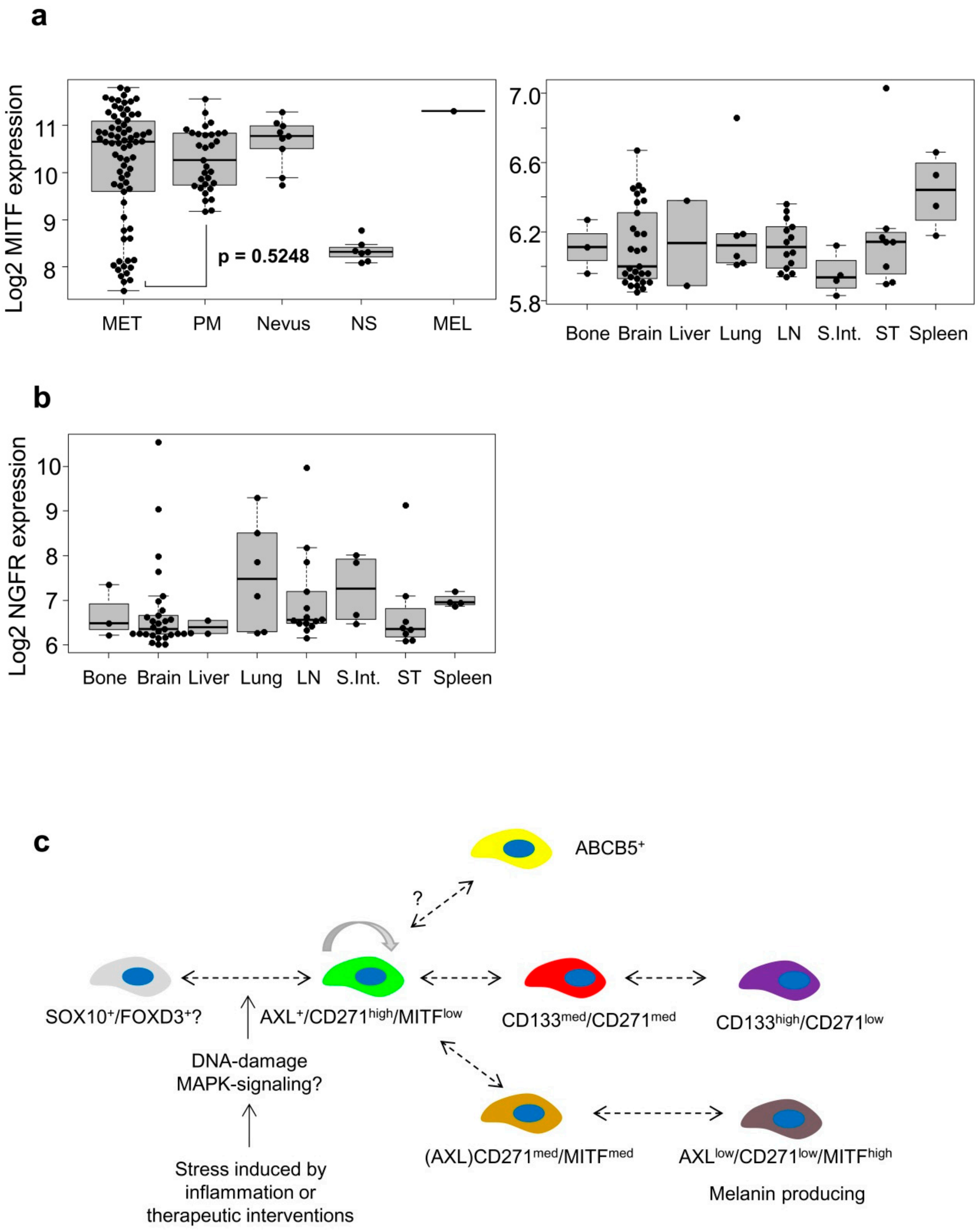

Figure S6. Phenotypical heterogeneity of melanoma. (**a**) Expression levels of MITF among melanoma progression (left bar chart) and among melanoma metastases indicate the presence of MITF^{high} and MITF^{low} tumors irrespective of the progression state of type of metastases. (**b**) Levels of CD271 expression among melanoma metastases indicate a potential prevalence in brain metastases (LN, lymph node; S.Int, small intestine; ST, soft tissue). (**c**) Phenotypic heterogeneity of melanoma cells. Phenotype switching and cellular plasticity define proliferative and invasive/migratory states of melanoma cells whereas high levels of AXL and CD271 define the invasive/migratory and high levels of MITF but low levels of AXL/CD271 define the proliferative state. Although MITF shows a mutually exclusive expression with AXL and CD271, cells may exist in intermediate states showing a

medium (med) expression of both. DNA damage, inflammation, and other stressors promote the AXL/CD271 state, which can give rise to CD133-expressing cells.

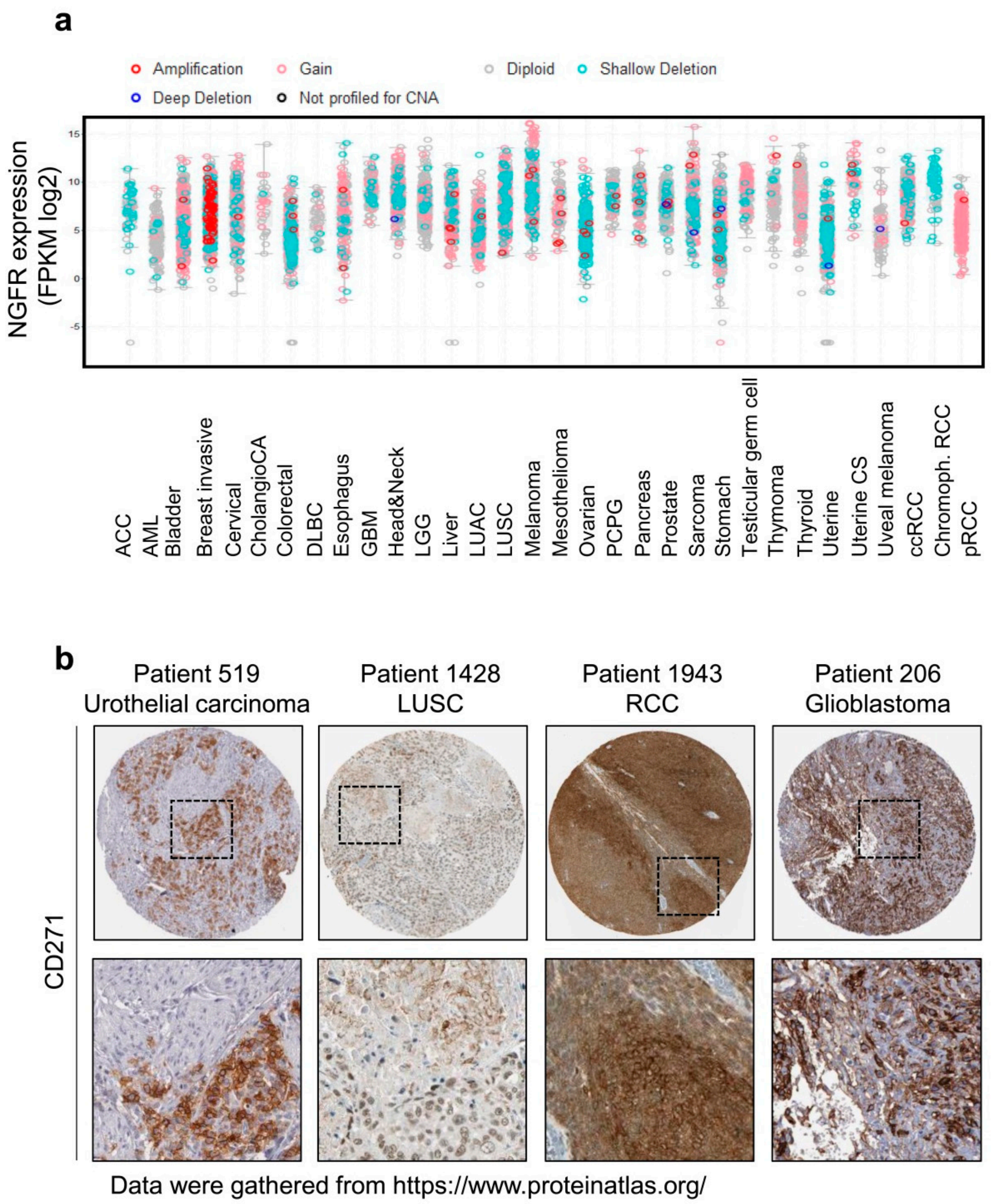

Figure S7. CD271 in cancer. (**a**) Presentation of CD271/NGFR expression data of Pan-TCGA studies copied from cBioportal showing tumors with amplified and lost expression of CD271. (**b**) Representative images of urothelial carcinoma, lung squamous cell carcinoma (LUSC), renal cell carcinoma (RCC), and gliobastoma stained with an CD271-specific antibody. Images where gathered from https://www.proteinatlas.org/.

Table S1. CD271 in other cancer types. Summary of cancer entities that comprise CD271-positive cellular subsets, the role of CD271 (if defined) in the respective entity, and associated pathways and prognostic value.

Cancer Type	Role of CD271	Pathways/partners known	Prognosis
**Clear Cell Renal Carcinoma**	CD271 and pro-BDNF promote cell survival, migration and invasion [1].	Pro-BDNF induces cell migration through CD271/TrkB/Sortilin and activation of MAPK/PI3K signaling.	High CD271: poor
**Head and Neck Squamous Cell Carcinoma (HNSCC)**	CD271 and CD44 define the tumor initiating cell population (HNSCC tumor-initiating cells). CD271 is expressed in most HNSCC, the knock down of CD271 inhibits proliferation via cell cycle arrest in the G2-M phase [2].<br>NGF-binding to CD271 confers enhanced invasion and metastasis through Slug expression [3].<br>Promotion of metastasis and resistance to anoikis through ADAM10-CD271-ICD-TRAF6-NFκB axis [4].	CD271 activation by NGF triggers MAPK signaling and drives EMT via up-regulation of *Snai2*/Slug, blocked by an CD271 neutralizing antibody [2,3].<br>CD271-ICD triggered EGFR-mediated autoubiquitination of TRAF6 [4].	High CD271: poor
**Hypopharyngeal Cancer**	Defines a stem cell-like population with tumor-initiating and repopulating capacity CD271$^+$ cells are located near CD34$^+$ vascular cells, at the invasion front and basal layer [5].<br>Initiates tumor formation by enhancing cell proliferation and accelerating migration [6].	Increased expression of Nanog and MMPs in CD271$^+$ cells.	High CD271: poor
**Oral Squamous Cell Carcinoma**	The expression of CD271 is found in basal layer cells of premalignant lesions and is significantly increased in malignant lesions, linked with tumor invasion and stemness as well as migration, metastasis and tumorigenicity [3,7,8].	Endocan/*ESM1* downstream target of CD271, regulates, proliferation, differentiation, migration, and cell adhesion [9].	High CD271: poor
**Lung Squamous Cell Carcinoma**	CD271 is increased and associated with aggressiveness in lung cancer squamous cell carcinoma, adenocarcinoma, SCLC) [10].<br>The knock down of CD271 suppressed, proliferation via arrest in G0 and decreased tumorigenicity and cell migration [11].	CD271 KD reduced MAPK activation [11].<br><br>TrkA-CD271-Sortilin [10].	High CD271: poor
**Glioblastoma**	Mediates invasion and resistance to therapy (chemo/radiation) in dependence to neurotrophins and binding to PDLIM1 [12–14].	PDLIM1 binds to the PDZ domain of CD271 [12].	High CD271: poor
**Thyroid Cancer**	CD271 is expressed in benign thyroid tissues and thyroid cancer and widely expressed in papillary thyroid carcinoma [15].	Cell survival and migration mediated by TrkA, CD271 and Sortilin [15].	CD271 is not a prognostic marker
**Breast Cancer**	CD271 overexpression favors tumor growth and survival via enhanced MAPK and BCL2 expression; increases cell survival via CD271-CTF (ADAM 17 mediated cleavage), inhibiting intrinsic apoptosis and increases resistance towards TRAIL [16–20]<br>CD271 defines a basal-like sub population with stem-like properties [21].<br>Promotion of metastasis and resistance to anoikis through ADAM10-CD271-ICD-TRAF6-NFκB axis [4]	CDKN1A/p21 and cIAP1 enhance survival and resistance to apoptosis in CD271$^+$ cells via NGF [16,18].<br>CD271-ICD triggered EGFR-mediated autoubiquitination of TRAF6 [4]	High CD271: poor
**Schwannoma**	CD271 is overexpressed in vestibular schwannomas	CD271 mediates resistance	NA

	compared to normal nerves and promotes cell survival by apoptosis resistance [22,23].	to apoptosis through pro-NGF and NFκB, independent of JNK [22,23].	
**Medulloblastoma**	CD271 mediates a reduced self-renewal capacity but increased sphere formation of medulloblastoma cells. The knock down of CD271 and gamma-secretase inhibitors decreased sphere formation [24,25].	CD271 is a specific and novel bio-marker of SHH-type medulloblastoma, targetable through MEK inhibition [24,25].	High CD271: favorable
**Esophageal Squamous Cell Carcinoma**	49.2% of ESCC specimens are CD271^{+}. Expressed on the infiltrative margins (Ki67^{+}) of differentiated tumors, diffusely distributed on undifferentiated tumors. CD271^{+} cells exhibit a high colony-forming capacity and resistance towards apoptosis [26].	CD271-TrKA	High CD271: favorable
**Gastric Cancer**	CD271 inhibits proliferation, invasion and metastasis, via down regulation of cyclins A/D1/E, MMP9 increase of TIMP1 and suppression of NFκB signaling [27,28].	CD271 suppresses MMP9 and NFκB signaling	High CD271: favorable
**Ewing Sarcoma**	https://cancerres.aacrjournals.org/content/78/13_Supplement/2074	NA	NA
**Osteosarcoma**	CD271 expression mediates stemness including self-renewal and tumorigenicity in generic osteosarcoma cell lines [29].	NA	NA

References

1. De la Cruz-Morcillo, M. A., J. Berger, R. Sanchez-Prieto, S. Saada, T. Naves, A. Guillaudeau, A. Perraud, P. Sindou, A. Lacroix, A. Descazeaud, et al. P75 neurotrophin receptor and pro-bdnf promote cell survival and migration in clear cell renal cell carcinoma. *Oncotarget* **2016**, 7, 34480–34497, doi:10.18632/oncotarget.8911.
2. Murillo-Sauca, O., M. K. Chung, J. H. Shin, C. Karamboulas, S. Kwok, Y. H. Jung, R. Oakley, J. R. Tysome, L. O. Farnebo, M. J. Kaplan, et al. Cd271 is a functional and targetable marker of tumor-initiating cells in head and neck squamous cell carcinoma. *Oncotarget* **2014**, *5*, 6854–6866, doi:10.18632/oncotarget.2269.
3. Chung, M. K., Y. H. Jung, J. K. Lee, S. Y. Cho, O. Murillo-Sauca, R. Uppaluri, J. H. Shin and J. B. Sunwoo. Cd271 confers an invasive and metastatic phenotype of head and neck squamous cell carcinoma through the upregulation of slug. *Clin. Cancer Res.* **2018**, *24*, 674–683, doi:10.1158/1078-0432.CCR-17-0866.
4. Bao, X., J. Shi, F. Xie, Z. Liu, J. Yu, W. Chen, Z. Zhang and Q. Xu. Proteolytic release of the p75(ntr) intracellular domain by adam10 promotes metastasis and resistance to anoikis. *Cancer Res.* **2018**, *78*, 2262–2276, doi:10.1158/0008-5472.CAN-17-2789.
5. Imai, T., K. Tamai, S. Oizumi, K. Oyama, K. Yamaguchi, I. Sato, K. Satoh, K. Matsuura, S. Saijo, K. Sugamura, et al. Cd271 defines a stem cell-like population in hypopharyngeal cancer. *PLoS One* **2013**, *8*, doi:e62002. 10.1371/journal.pone.0062002.
6. Mochizuki, M., K. Tamai, T. Imai, S. Sugawara, N. Ogama, M. Nakamura, K. Matsuura, K. Yamaguchi, K. Satoh, I. Sato, et al. Cd271 regulates the proliferation and motility of hypopharyngeal cancer cells. *Sci. Rep.* **2016**, *6*, 30707, doi10.1038/srep30707.
7. Kiyosue, T., S. Kawano, R. Matsubara, Y. Goto, M. Hirano, T. Jinno, T. Toyoshima, R. Kitamura, K. Oobu and S. Nakamura. Immunohistochemical location of the p75 neurotrophin receptor (p75ntr) in oral leukoplakia and oral squamous cell carcinoma. *Int. J. Clin. Oncol.* **2013**, *18*, 154–163, doi:10.1007/s10147-011-0358-4.
8. Tong, D., J. Sun, P. Huang, M. Li and F. Zhang. P75 neurotrophin receptor: A potential surface marker of tongue squamous cell carcinoma stem cells. *Mol. Med. Rep.* **2017**, *15*, 2521–2529, doi:10.3892/mmr.2017.6291.

9. Chen, C., J. H. Shin, J. T. Eggold, M. K. Chung, L. H. Zhang, J. Lee and J. B. Sunwoo. Esm1 mediates ngfr-induced invasion and metastasis in murine oral squamous cell carcinoma. *Oncotarget* **2016**, *7*, doi:70738–70749, 10.18632/oncotarget.12210.
10. Gao, F., N. Griffin, S. Faulkner, C. W. Rowe, L. Williams, S. Roselli, R. F. Thorne, A. Ferdoushi, P. Jobling, M. M. Walker, et al. The neurotrophic tyrosine kinase receptor trka and its ligand ngf are increased in squamous cell carcinomas of the lung. *Sci. Rep.* **2018**, *8*, 8135, doi:10.1038/s41598-018-26408-2.
11. Mochizuki, M., M. Nakamura, R. Sibuya, T. Okazaki, J. Abe, T. Nakagawa, S. Takahashi, T. Yamazaki, T. Imai, A. Takano, et al. Cd271 is a negative prognostic factor and essential for cell proliferation in lung squamous cell carcinoma. *Lab. Invest.* **2019**, *99*, 1349–1362, doi:10.1038/s41374-019-0246-5. https://doi.org/10.1038/s41374-019-0246-5.
12. Ahn, B. Y., R. F. Saldanha-Gama, J. J. Rahn, X. Hao, J. Zhang, N. H. Dang, M. Alshehri, S. M. Robbins and D. L. Senger. Glioma invasion mediated by the p75 neurotrophin receptor (p75(ntr)/cd271) requires regulated interaction with pdlim1. *Oncogene* **2016**, *35*, 1411–1422, doi:10.1038/onc.2015.199.
13. Johnston, A. L., X. Lun, J. J. Rahn, A. Liacini, L. Wang, M. G. Hamilton, I. F. Parney, B. L. Hempstead, S. M. Robbins, P. A. Forsyth, et al. The p75 neurotrophin receptor is a central regulator of glioma invasion. *PLoS Biol.* **2007**, *5*, e212, doi:10.1371/journal.pbio.0050212.
14. Alshehri, M. M., S. M. Robbins and D. L. Senger. The role of neurotrophin signaling in gliomagenesis: A focus on the p75 neurotrophin receptor (p75(ntr)/cd271). *Vitam. Horm.* **2017**, *104*, 367–404, doi:10.1016/bs.vh.2016.11.001.
15. Faulkner, S., P. Jobling, C. W. Rowe, S. M. Rodrigues Oliveira, S. Roselli, R. F. Thorne, C. Oldmeadow, J. Attia, C. C. Jiang, X. D. Zhang, et al. Neurotrophin receptors trka, p75(ntr), and sortilin are increased and targetable in thyroid cancer. *Am. J. Pathol.* **2018**, *188*, 229–241, doi:10.1016/j.ajpath.2017.09.008.
16. Verbeke, S., S. Meignan, C. Lagadec, E. Germain, H. Hondermarck, E. Adriaenssens and X. Le Bourhis. Overexpression of p75(ntr) increases survival of breast cancer cells through p21(waf1). *Cell. Signal.* 2010, 22, 1864–1873, doi:10.1016/j.cellsig.2010.07.014.
17. Verbeke, S., E. Tomellini, F. Dhamani, S. Meignan, E. Adriaenssens and B. Xuefen le. "Extracellular cleavage of the p75 neurotrophin receptor is implicated in its pro-survival effect in breast cancer cells." *FEBS Lett.* **2013**, *587*, 2591–2596, doi:10.1016/j.febslet.2013.06.039.
18. Descamps, S., V. Pawlowski, F. Revillion, L. Hornez, M. Hebbar, B. Boilly, H. Hondermarck and J. P. Peyrat. Expression of nerve growth factor receptors and their prognostic value in human breast cancer. *Cancer Res.* **2001**, *61*, 4337–4340.
19. Descamps, S., R. A. Toillon, E. Adriaenssens, V. Pawlowski, S. M. Cool, V. Nurcombe, X. Le Bourhis, B. Boilly, J. P. Peyrat and H. Hondermarck. Nerve growth factor stimulates proliferation and survival of human breast cancer cells through two distinct signaling pathways. *J. Biol. Chem.* **2001**, *276*, 17864–17870, doi:10.1074/jbc.M010499200.
20. Deng, X., G. Xu, L. He and M. Xu. P75ntr promotes survival of breast cancer resistant cells by regulating bcl-2/bax and mapk pathway. *Int. J. Clin. Exp. Pathol.* **2017**, *10*, 11685–11694.
21. Kim, J., R. Villadsen, T. Sorlie, L. Fogh, S. Z. Gronlund, A. J. Fridriksdottir, I. Kuhn, F. Rank, V. T. Wielenga, H. Solvang, et al. Tumor initiating but differentiated luminal-like breast cancer cells are highly invasive in the absence of basal-like activity. *Proc. Natl. Acad. Sci. USA* **2012**, *109*, 6124–6129, doi:10.1073/pnas.1203203109.
22. Gentry, J. J., P. Casaccia-Bonnefil and B. D. Carter. Nerve growth factor activation of nuclear factor kappab through its p75 receptor is an anti-apoptotic signal in rn22 schwannoma cells. *J. Biol. Chem.* **2000**, *275*, 7558–7565, doi:10.1074/jbc.275.11.7558.
23. Ahmad, I., W. Y. Yue, A. Fernando, J. J. Clark, E. A. Woodson and M. R. Hansen. P75ntr is highly expressed in vestibular schwannomas and promotes cell survival by activating nuclear transcription factor kappab. *Glia* **2014**, *62*, 1699–1712, doi:10.1002/glia.22709.
24. Liang, L., C. Aiken, R. McClelland, L. C. Morrison, N. Tatari, M. Remke, V. Ramaswamy, M. Issaivanan, T. Ryken, M. R. Del Bigio, et al. Characterization of novel biomarkers in selecting for subtype specific medulloblastoma phenotypes. *Oncotarget* **2015**, *6*, 38881–3900, doi:10.18632/oncotarget.6195.
25. Liang, L., L. Coudiere-Morrison, N. Tatari, M. Stromecki, A. Fresnoza, C. J. Porter, M. R. Del Bigio, C. Hawkins, J. A. Chan, T. C. Ryken, et al. Cd271(+) cells are diagnostic and prognostic and exhibit elevated mapk activity in shh medulloblastoma. *Cancer Res.* **2018**, *78*, 4745–4759, doi:10.1158/0008-5472.CAN-18-0027.

26. Okumura, T., S. Tsunoda, Y. Mori, T. Ito, K. Kikuchi, T. C. Wang, S. Yasumoto and Y. Shimada. The biological role of the low-affinity p75 neurotrophin receptor in esophageal squamous cell carcinoma. *Clin. Cancer Res.* **2006**, *12*, 5096–5103, doi:10.1158/1078-0432.CCR-05-2852.
27. Jin, H., Y. Pan, L. He, H. Zhai, X. Li, L. Zhao, L. Sun, J. Liu, L. Hong, J. Song, et al. P75 neurotrophin receptor inhibits invasion and metastasis of gastric cancer. *Mol. Cancer Res.* **2007**, *5*, 423–433, doi:10.1158/1541-7786.MCR-06-0407.
28. Jin, H., Y. Pan, L. Zhao, H. Zhai, X. Li, L. Sun, L. He, Y. Chen, L. Hong, Y. Du, et al. P75 neurotrophin receptor suppresses the proliferation of human gastric cancer cells. *Neoplasia* **2007**, *9*, 471–478, doi:10.1593/neo.07175.
29. Tian, J., X. Li, M. Si, T. Liu and J. Li. Cd271+ osteosarcoma cells display stem-like properties. *PLoS One* **2014**, *9*, e98549. doi:10.1371/journal.pone.0098549.

© 2020 by the authors. Submitted for possible open access publication under the terms and conditions of the Creative Commons Attribution (CC BY) license (http://creativecommons.org/licenses/by/4.0/).
